# Characterization of the Acid-Base Character of Burned Clay Ceramics Used for Water Decontamination

**DOI:** 10.3390/ma12233836

**Published:** 2019-11-21

**Authors:** Andrei Victor Sandu, Viorica Vasilache, Ioan Gabriel Sandu, Joseph M. Sieliechi, Innocent Kouassi Kouame, Petre Daniel Matasaru, Ion Sandu

**Affiliations:** 1 Faculty of Materials Science and Engineering, Gheorghe Asachi Technical University of lasi, Blvd. D. Mangeron 71, 700050 lasi, Romania; 2 Center of Excellence Geopolymer & Green Technology (CeGeoGTech), School of Material Engineering, Universiti Malaysia Perlis (UniMAP), P. O. Box 77, d/a Pejabat Pos Besar, 01000 Kangar, Perlis Malaysia; 3 Arheoinvest Centre, Institute of Interdisciplinary Research – Department of Science, Alexandru Ioan Cuza University, 11 Carol I Blvd, 700506 Iasi, Romania; viorica_18v@yahoo.com (V.V.); ion.sandu@uaic.ro (I.S.); 4 National School of Agro Industrial Sciences (ENSAI), University of Ngaoundere-Ngaoundere, PO.Box 455 00237 Ngaoundere, Cameroon; jsieliechi@yahoo.fr; 5 Laboratory of Geosciences and Environment, UFR Science and Environmental Management, Nangui Abrogoua University, 28 BP 847 28 Abidjan, Ivory Coast; innocent_kouassi@yahoo.fr; 6 Telecommunications and Information Technology, Faculty of Electronics, Gheorghe Asachi Technical University of lasi, Carol I 11A, 700506 lasi, Romania; 7 Romanian Inventors Forum, Str. Sf. P. Movila 3, 700089 Iasi, Romania

**Keywords:** ceramics, acid-base character, caustic module, water treatment, SEM-EDX

## Abstract

The paper presents the results of ample investigations performed on industrial and traditional ceramics of fired clay used in processes of water potabilization in the last stage of filtration, after that of active charcoal. Using the data obtained through the scanning electron microscope coupled with energy dispersive X-ray analysis (SEM-EDX) and pH analyses, on the basis of the atomic composition and free concentration of hydronium ions, the normal caustic (Si/Al) and summative [(Si+Ti+FeIII+Cl)/(Al+Ca+Mg+Na+K)] modules were assessed, which were correlated with the free acidity and, respectively, the capacity of absorption and ionic exchange of the Fe^3+^ and Al^3+^ ions. The study allowed the selection, on the basis of the caustic module, of the ceramics with high capacity for ionic exchange.

## 1. Introduction

The capacity for ionic exchange is due to the acidic marginal structures of the type Si(IV)–O–H+ and of the hydroxide ones Al(III)–OH. The base-acidic activity of these groups from the structure of the fired-clay ceramics is due to the Si:Al stoichiometric ratio (known in practice as the caustic module, which varies from 1:1 to 4:1), but also to the position of the two coordination centers of the basic elementary cell. It is known that the basic units of fired-clay ceramics are tetrahedrons of silicate anions and octahedrons of aluminate anions, connected by piro-links in various stratified or coplanar two-dimensional and, respectively, three-dimensional structural forms, which at the surface, present hydroxide (HO–) groups, amphoteric, with double-linked oxygen and acidic structures (–O–H+). Generally, because of the different mineralogical nature of the base clay (composed of kaolinite, montmorillonite, chlorite, halloysite, illite, vermiculite, smectite, pyrophyllite, etc.) and of the firing technology, but also the foundations, plasticizers and other additives employed in the manufacturing, the acid-basic character, given by the marginal groups, varies widely, from weakly acidic (pH ≤ 4,5) to weakly alkaline (pH ≤ 9,5) [1,2,3,4,5,6]. An important role in the capacity for ionic exchange is that of the porosity and, respectively, the active surface area of the granulite’s. The capacity for ionic exchange is, thus, directly proportional to the capacity of absorption and the active surface.

The number and distribution of groups capable of ionic exchange is given both by the mineralogical composition of the raw materials, the composition before the plastic and thermal processing, as well as the conditions of calcination, vitrification, and glazing, which have variable parameters: heating speed, firing temperature, firing duration, firing system (closed, open or semi-open), with a direct or hidden source, oxidative, or reducing, etc. [6,7,8,9].

The capacity for ionic exchange is due only to the acidic structures Si(IV)–O^–^H^+^, Ti(IV)–O^–^H^+^, and Fe(III)–O^–^H^+^. The ceramics with high concentrations of Al(III), Ca(II), and Mg(II) have a character that varies from amphoteric to weakly basic, while those with Si(IV), Ti(IV), and Fe(III) vary from amphoteric to acidic [10,11,12,13,14,15,16].

The main advantage of ceramic products used in treating and purifying water is due to the mechanic resistance, the acid-basic and redox stability, but also to the thermal and photochemical stability, as well as to an extensive series of very important characteristics, such as the apparent density or specific weight, under 1500 kg/m^3^, and compression resistance, which varies between 50 and 200 daN/cm^2^; the maximum quantity of absorbed water, which varies between 8% and 20%; the gelifraction or the phenomenon of mechanical deterioration (breakup) of the products saturated with water due to freezing and thawing, then their biodegradation and biodeterioration through biochemical and chemical processes, due to the specific loads of the treated waters. Likewise, another advantage of the industrial ceramics is given by the minimum concentration of soluble components in aqueous systems [17,18,19,20,21,22,23,24,25,26,27,28,29].

For this reason, the granules used in water treatment are beforehand subjected to cleaning processes by dispersion in containers with deionized—weakly acidic water in which they are lightly agitated for several tens of minutes, after which the water is decanted and the ceramics are dried with warm air [6].

The aim of this study is to assess the acid-basic character in correlation with the scanning electron microscopy coupled with energy dispersive X-Ray Spectroscopy (SEM-EDX), the free acidity, and, respectively, to assess the capacity for absorption and ionic exchange of the cations Fe^3+^ and Al^3+^, of the fired-clay ceramics originating from industrial bricks and traditional pots. The study allowed the selection of the ceramics optimal for eliminating the traces of Fe^3+^ and Al^3+^, of the taste and smell of the treated waters. The ceramics will be employed in the final filtration stage of the treatment of underground and surface waters in order to make them drinkable, producing quality water with impressive organoleptic characteristics.

## 2. Experimental

### 2.1. Ceramics Under Consideration 

The research focused on two types of ceramics:

- *Industrial ceramics*, labelled CI, in the form of granules from freshly-fired bricks that were crushed;
- *Traditional ceramics*, labelled CT, in the form of granules obtained by crushing the neck of glazed pottery, freshly fired.

Three groups of samples were collected from the two sample groups, separated using a granulometric sieve with openings of 1.5–2.5 mm, 2.5–3.5 mm, and, respectively, 3.5–6.5 mm. The resulting samples were stored in plastic containers with screwed caps.

### 2.2. Processing and Analyzing the Samples

The composition of each lot was determined by collecting small chips weighing ca. 10–20g, with two parallel surfaces and indexed as following:

- CI_a_, acidic industrial ceramics;
- CI_sa_, weakly-acidic industrial ceramics;
- CI_am_, amphoteric industrial ceramics;
- CI_saHCl_, weakly-acidic industrial ceramics treated with HCl 3M;
- CI_amHCl_, amphoteric industrial ceramics treated with HCl 3M;
- CT_a_, acidic traditional ceramics;
- CT_sa_, weakly-acidic traditional ceramics;
- CT_amsa_, amphoteric to weakly-acidic traditional ceramics;
- CT_am_, amphoteric traditional ceramics;
- CT_amsb_, weakly-basic amphoteric traditional ceramics;
- CT_amHCl_, amphoteric traditional ceramics treated with HCl 3M;
- CT_amsbHCl_, weakly acidic amphoteric traditional ceramics treated with HCl 3M.

These samples were analyaed from the point of view of elemental chemical composition and internal structure by means of scanning electron microscopy coupled with X-ray spectrometry (SEM-EDX). The equipment used for this stage consisted of an SEM VEGA II LSH scanning electron microscope produced by Tescan (Brno, Czech Republic) and a Quantax QX2 EDX detector produced by Bruker/Roentec (Berlin, Germany).

For each sample, we determined the free acidity and, respectively, the pH of the solution resulting from dispersing 90 g of finely grounded (1.5–2.5 mm fraction separated using the granulometric sieve) material from each sample into 100 mL of twice-distilled water. The resulting data were correlated with the elemental composition provided by the EDX.

The free acidity was determined from the value of the pH when the granules with a diameter of 1.5–2.5 mm were dispersed in the twice-distilled water, namely:

[H^+^] = 10^−pH^, moles/L.

### 2.3. Evaluation of retention capacity 

To evaluate the capacity of retention of ions of Fe^3+^ and Al^3+^ for those two groups of ceramics three sets of samples (1.5–2.5mm; 2.5–3.5mm and 3.5–6.5mm granulometry) were involved. The granules were indexed as follow:

- CI_m_, control industrial ceramic (untreated with aqueous solution of chloride acid for solubilization of labile components) having granulometry: CI_m_(1.5–2.5); CI_m_(2.5–3.5); CI_m_(3.5–6.5);
- CI_HCl_, industrial ceramic treated with aqueous solution of chloride acid 5M, having granulometry: CI_HCl_(1.5–2.5); CI_HCl_(2.5–3.5); CI_HCl_(3.5–6.5);
- CT_m_, control traditional ceramic (untreated), having granulometry: CT_m_(1.5–2.5); CT_m_(2.5–3.5); CT_m_(3.5–6.5);
- CT_HCl_, treated traditional ceramic (aqueous solution of HCl 5M), having granulometry CT_HCl_(1.5–2.5); CT_HCl_(2.5–3.5); CT_HCl_(3.5–6.5).

The capacity of retention of ions of Fe^3+^ and Al^3+^ from aqueous solutions of 0.5 M was performed by dispersion in 100 mL water of 90 g ceramic granules, under weak agitation at room temperature for 20 min. Before dispersion, ceramic granules were washed in bi-distilled water, dried for 4 h at 105 °C in an oven. After 20 min of dynamic dispersion, residual iron quantity was determined by atomic absorption spectroscopy. The retaining capacity was calculated by the formula: CR = (C_r_/C_i_)×100%, where CR is the retaining capacity (%), C_r_—is the final concentration (mol/L), and C_i_—initial concentration (mol/L).

## 3. Results and Discussions 

### 3.1. Determining the Chemical Nature and the Physical Microstructures of the Ceramics

**Figure 1:**
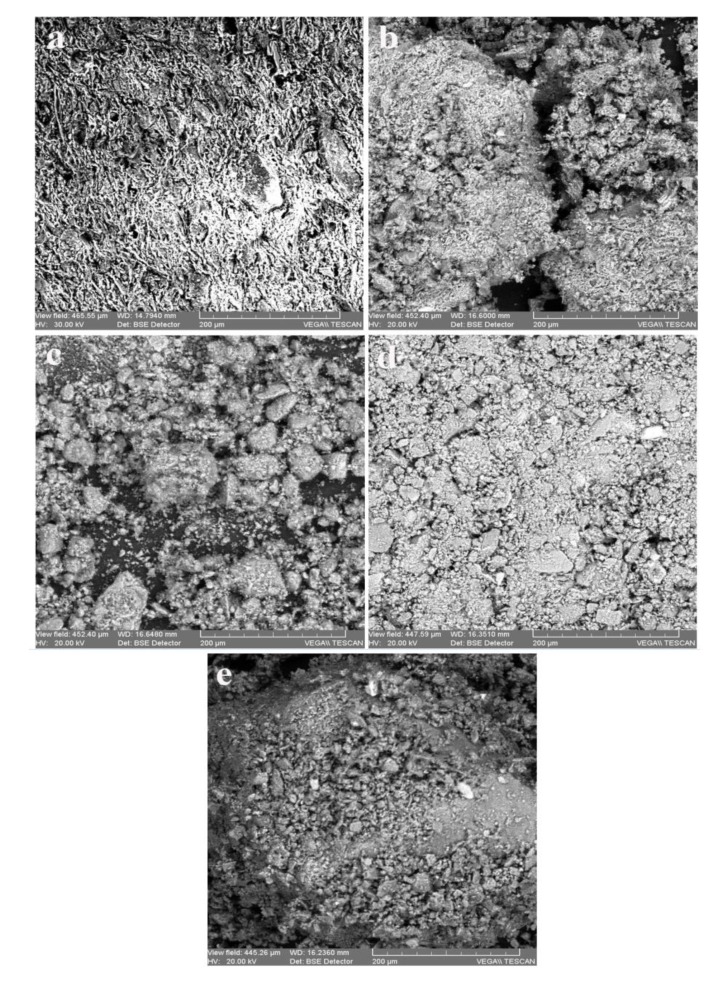
Scanning electron microscope images of the industrial ceramic samples: (**a**) CI_a_ acidic ceramic; (**b**) CI_sa_ weakly-acidic ceramic; (**c**) CI_am_ amphoteric ceramic; (**d**) CI_saHCl_ weakly-acidic ceramic treated with HCl 3M; (**e**) CI_amHCl_ amphoteric ceramic treated with HCl 3M

**Figure 2:**
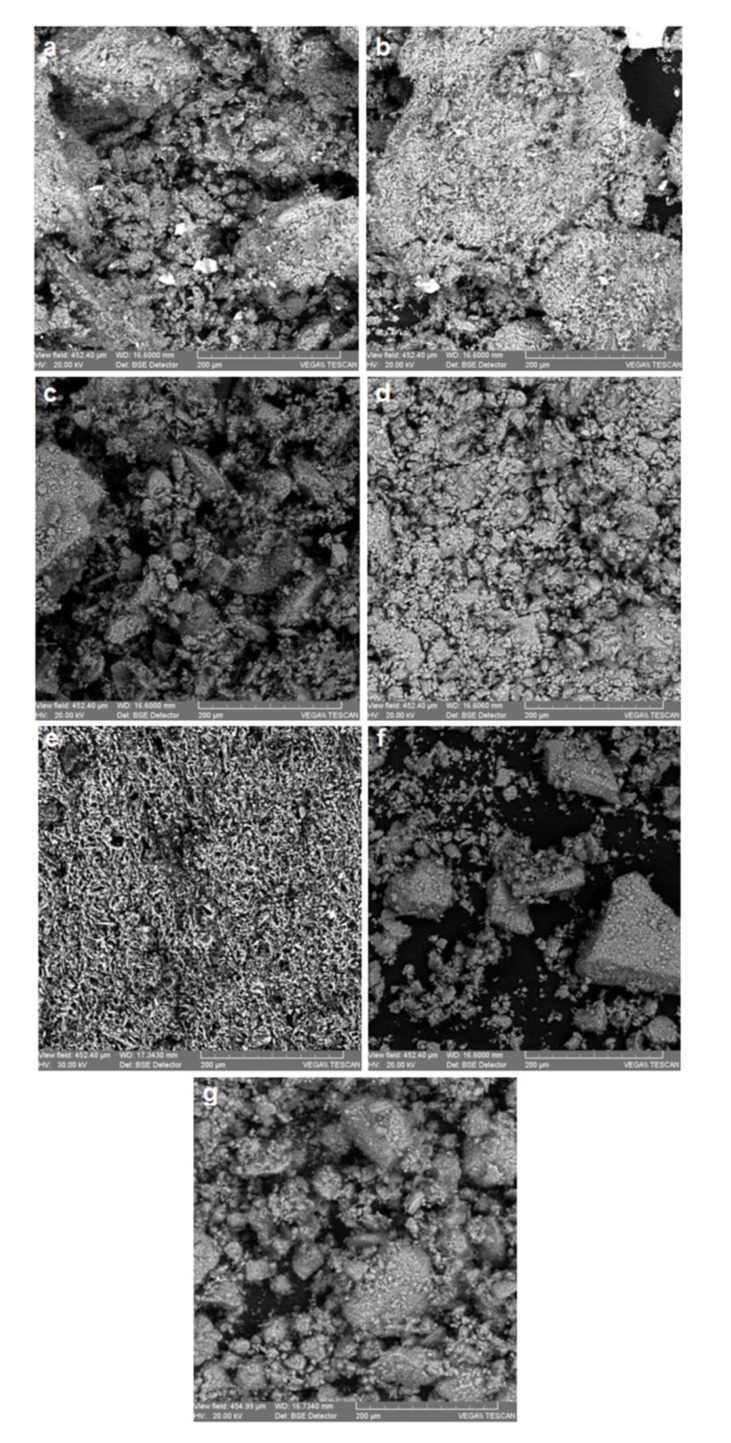
Scanning electron microscope images of the traditional ceramic samples (CT): (**a**) CT_a_ acidic ceramic; (**b**) CT_sa_ weakly-acidic ceramic; (**c**) CT_amsa_ amphoteric low-acidic ceramic; (**d**) CT_amsb_ amphoteric low-alkaline ceramic, (**e**) CT_am_ amphoteric ceramic, **f**) CT_amHCl_ amphoteric ceramic treated with HCl 3M, (**g**) CT_amsbHCl_ amphoteric low-alkaline ceramic treated with HCl 3M.

Figure 1 and Figure 2 presents the SEM microphotographs of the 12 samples analyzed (five from industrial bricks and seven from traditional pots).

The SEM analyzes allow, on the basis of the microphotographs from Figure 1 and Figure 2, assessing the structure and morphology of the sintering granules of the ceramic material.

The industrial ceramics display in the volume phase a finer granulation and the somewhat uniform distribution of the acidic alumino-silicate granulite’s, whereas the traditional ceramics have a coarse and nonhomogeneous granulation, in which besides the acidic base structures there are often fragments of glazing and basic glasses.

Table 1 presents the elemental composition of the 12 ceramics under analysis.

The data from the energy dispersive X-ray (EDX) elemental analysis from Table 1 confirms the high compositional similarity of the industrial ceramics, explainable by the rigorously controlled dosing of the bisque or paste ingredients, whereas in the case of the traditional ceramics, the compositions vary widely for the same and between potters, who moreover used different clays and compositions for producing the bisque.

It must be recalled that initially, a much higher number of samples were advanced for these analyses, both from industrial ceramics (20) and traditional wares (30), from which only the most representative, in terms of chemical composition as ascertained from the SEM-EDX analyses, were selected.

The data from the EDX elementary analysis (Table 1) allow correlations between the value of the normal caustic module and that of the summative module and the free acidity. It must be stressed that in the case of the free acidity, an important role is played by the Cl^−^ ion, native, or induced from the HCl treatment.

The basic module of a ceramic structure that determines its acid-base behavior is given by the Si(IV)/Al(III) ratio. With respect to the three-dimensional structure of a ceramic, we speak of structures with *acidic functions* (titanium [TiO_4_] octahedrons or tetrahedrons), interspersing those of silicate [SiO_4_] and aluminate [AlO_3_], which are joined by the groups with *basic functions* (Ca–OH, Mg–OH, etc.) that co-act in the acid-base behavior. The exception is the marginal ions Cl^−^ and S^2−^, which have dominant acidic function.

Table 2 presents the values of the normal caustic Si/Al modules, and the summative Si(Ti/Fe^III^+Cl)/Al(Ca/Mg+Na/K) of the 12 types of ceramics, which are correlated with their free acidity.

### 3.2. The Free Acidity of the Ceramics 

The free acidity of the samples listed above and, respectively, the pH of the solution resulting from dispersing 90 g of finely grounded (1.5–2.5 mm fraction separated using the granulometric sieve) material from each sample into 100 mL of twice-distilled water. The resulting data were correlated with the elemental composition provided by the EDX. 

Table 2 presents the correlation between the normal caustic and summative modules, and the free acidity, expressed through the value of the pH obtained by dispersing the 1.5–2.5 mm granules into the twice-distilled water and, respectively, the [H^+^] concentration calculated by the formula:

[H^+^] = 10^−pH^, moles/L.

The resulting data show a high correlation between the normal caustic module and the summative one, obtained on the basis of the EDX atomic composition of the 12 types of ceramics. The data allowed sorting the 12 types of untreated and treated ceramics according to the decrease in the free acidity.

The present study allowed selecting the optimal ceramics for use in processes of chemo-absorption from the final stage of filtering ground and surface waters in order to make them drinkable, respectively, to obtain pure water with high organoleptic characteristics. In this regard, of interest are the ceramics with the caustic module between 1.1 and 2.8.

These ceramics have been featured in a new procedure for water potabilization using current city wastewater plants [6].

### 3.3. Retention Capacity of Fe^3+^ and Al^3+^ Ions

To evaluate the capacity of retention of ions Fe^3+^ and Al^3+^ of two types of considered granules, using only acid ceramics, CI_a_ and CT_a_, after fine crushing the samples were separated in sieves having stitches of: 1.5–2.5mm; 2.5–3.5mm and 3.5–6.5mm, and indexed as follow:

- *Control industrial ceramic*, granulometry: CI_m_(1.5–2.5); CI_m_(2.5–3.5); CI_m_(3.5–6.5);
- *Industrial ceramic treated* with solution of HCl 5M, granulometry CI_HCl_(1.5–2.5); CI_HCl_(2.5–3.5); CI_HCl_(3.5–6.5);
- *Control traditional ceramic*, granulometry: CT_m_(1.5–2.5); CT_m_(2.5–3.5); CT_m_(3.5–6.5);
- *Traditional ceramic treated* with solution of HCl 5M, granulometry CT_HCl_(1.5–2.5); CT_HCl_(2.5–3.5); CT_HCl_(3.5–6.5).

**Figure 3:**
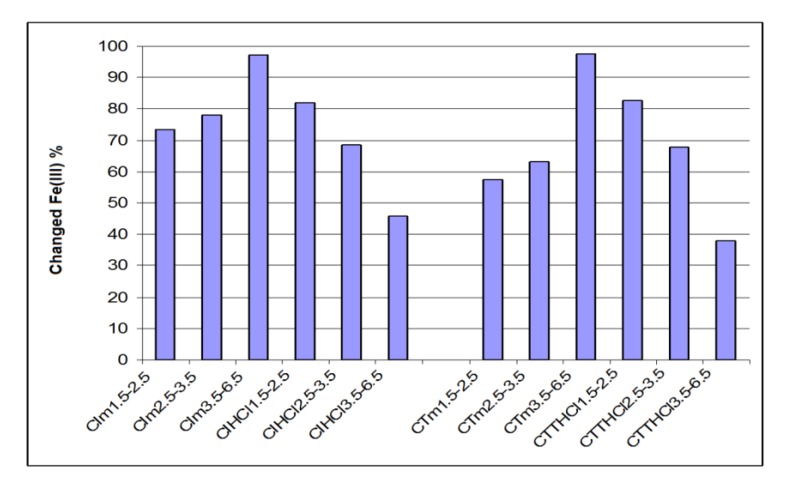
Iron percent changed through chemo-sorption in industrial and traditional ceramics treated with HCl and untreated.

**Figure 4:**
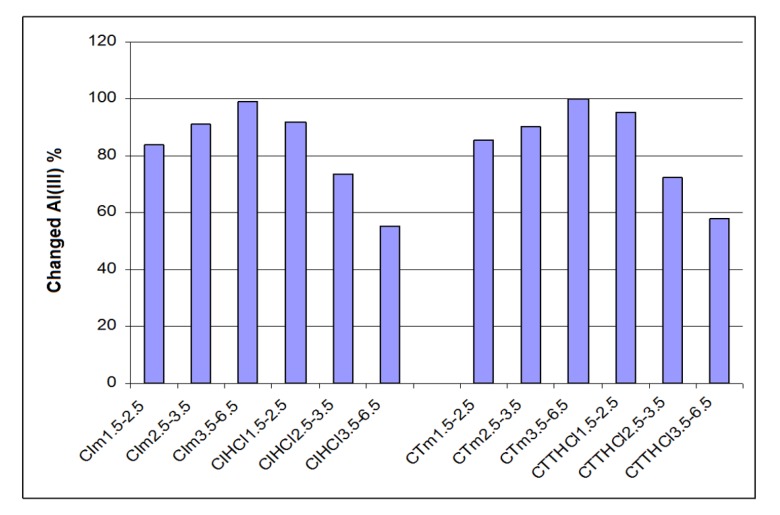
Aluminum percent changed through chemo-sorption in industrial and traditional ceramics treated with HCl and untreated.

Figure 3 and Figure 4 present the retaining capacity of ions Fe^3+^ and Al^3+^ by percent of iron and aluminum changed by chemo-sorption for analyzed ceramics, function of three granulometric groups (for industrial ceramics CI and traditional ceramics CT), as such or treated with an aqueous solution of HCl 3 M.

Conforming of data from Figure 3, the change in iron percent of traditional ceramics treated with a solution of HCl 3 M decreased with increasing granulometry, so for granulometry of 1.5–2.5 mm, compared to 3.5–6.5 mm, the change in iron percent decreased from 81.9% to 45.6%. Tendency is inverted for the untreated traditional ceramics, where increases in iron percent were directly proportional with granulometry. So, for granulometry of 1.5–2.5 mm, comparing to 3.5–6.5mm, percent of the changed iron increase was proportional from 73.5% to 97.2%.

The same tendency was observed for industrial ceramic. Treated industrial ceramic presented for granulometry of 1.5–3.6 mm comparing to 3.5–6.5 mm a percent of changed iron in decreasing from 82.7% to 37.9%, while for the untreated one, the effect was inverted, the values of the change in iron percent increased from 57.5% to 97.5%.

Similar, the data from Figure 4 prove that for traditional ceramics treated with HCl 3 M, the percent of changed aluminum decreased when granulometry increased, so for granulometry of 1.5–2.5 mm, compared with 3.5–6.5 mm, the percent of changed aluminum decreased from 92.1% to 55.3%. The tendency is inverted for untreated traditional ceramic because the percent of changed aluminum increase was directly proportional with granulometry. So, for granulometry of 1.5–2.5 mm, compared to 3.5–6.5 mm, the percent of changed aluminum increased proportionally from 83.8% to 99.2%.

The same tendency was noted for industrial ceramic. So, treated industrial ceramic presented a granulometry of 1.5–3.6 mm compared to 3.5–6.5 mm, a decreasing percent of changed iron from 95.2% to 57.8%, while for those untreated, the effect was inverted, the percentage change in aluminum increased from 85.7% to 99.8%.

Initially, the capacity of ionic change for five industrial and seve traditional ceramics was studied, and it was observed that only those acids (CI_a_ and CT_a_) presented a good reproducibility of the results. These ceramics were used for ionic change of other cations, considered as dangerous to surpass the limit levels of toxicity, like Hg^2+^, Pb^4+^, Pb^3+^, Ni^2+^, As^3+^, etc., but of which results did not present reproducibility similar to Fe^3+^ and Al^3+^. This fact is caused by the acid-base and redox action of ceramics in the caustic module, with polyhedron structures Si(IV), Ti(IV), and Fe(III) which confer a redoxite behavior with detoxification of some molecular structures and retention in potabilization process, as molecular chlorine reduced to chloride ion and a serial of cations (Hg^2+^, Pb^3+^, Pb^4+^, As^5+^, etc.), which are reduced to hardly soluble structures (Hg(I), Pb(II), As(III), etc.) retained in the ceramic. A cumulative effect was not be observed.

This domain is very interesting for science and technology; therefore, deeper research must be conducted. This study allowed the selection of optimum ceramics which could be used for chemiorption processes in the final stage of filtering and treating of ground and surface waters. These ceramics were the base to elaborate a new method of potabilization of waters [6].

The resulted data were re-validated using statistical, probabilistic, and numerical methods for analysis, keeping in mind future research involving IoT-based methods for the analysis.

## 4. Conclusions

As stated by the specialized literature, the capacity for ionic exchange of the normal fired-clay ceramics is due to the acidic structures of the type Si(Ti)–O^–^H^+^. The capacity for ionic exchange is influenced by the caustic module (Si/Al ratio), the porosity, and active surface of the ceramic granules. The number and distribution of grouping capable of ionic exchange is given, on the one hand, by the mineralogical composition of the raw materials, and the composition of the mixture before the plastic processing and the firing, and, on the other hand, by the conditions of calcination and vitrification, with their variable parameters—heating speed, firing temperature, firing duration, firing system (closed, open, semi-open), source type (direct or hidden), firing type (oxidative or reducing), etc.

The present study was carried out on two lots of freshly-produced ceramics, namely industrial bricks and traditional pots, in order to select the optimal type, from the acid-base point of view, for use in chemo-absorption processes during the final filtration stage of ground and surface water potabilization treatment, allowing the following conclusions. 

The normal ceramic materials used in treating and purifying waters have, compared to other filtration materials, a series of advantages, including good mechanical resistance, acid-basic, and redox chemical stability, thermal and photochemical stability, but also an extensive series of very important characteristics for the purpose at hand, such as the apparent density or specific weight under 1500 Kg/m^3^ and resistance to compression, which varies between 50 and 200 daN/cm^2^; the maximum quantity of water absorbed (between 8% and 20%); gelifraction, or mechanical deterioration (breaking), and degradation of products saturated with water under the action of freezing and thawing and the biochemical and chemical processes, under the influence of the specific composition of the waters undergoing treatment, and last but not least, the low price.

Conforming with data from ionic change for ion Fe^3+^, it was noted that for traditional ceramics treated with HCl 3 M, the percent of change in iron decreased when granulometry increased; This tendency is inverted for untreated traditional ceramic where the percentage of changed iron increased when granulometry increased.

The same tendency was noted for industrial ceramic. Treated industrial ceramic exhibit an decreasing of percent of changed iron when granulometry increase, while for the untreated ceramic, an increasing of percent of changed iron when granulometry increase;

Regarding the percent of changed aluminum, for traditional ceramics treated with HCl 3M, this decrease when granulometry increase, and the behavior inverted for untreated ceramics, when the percent of changed aluminum increase when granulometry increase; The same tendency was deduced for treated industrial ceramic, when the percent of changed aluminum decreased with increased granulometry, while for untreated industrial ceramics the percentage of changed aluminum increased when granulometry increased.

The study allowed selecting ceramics with optimum properties regarding the ionic change, able to be used in the final stage of filtering and treating the process of ground and surface waters to obtain fresh potable water. The better results exhibit acid granules notes as CI_a_ and CT_a_;

The ceramics with the caustic module between 1.1 and 2.8 can be used for the ionic exchange of other cations that risk exceeding the threshold for toxicity. 

As a general conclusion, these materials are very good to be used in water treatment due to their low cost, being waste materials or scraps from the technological flow of construction bricks/tiles or traditional ceramics. The resulting water has good organoleptic properties.

## Figures and Tables

**Table 1:** Chemical elemental composition of the studied ceramics.

Sample	Elemental Composition, Atomic Percentages (at %)									
	Si	Al	Fe	Ca	Mg	K	Na	Cl	Ti	O
CI_a_	23.685	6.085	3.948	3.555	2.320	1.998	1.238	–	0.560	56.611
CI_sa_	16.604	6.541	2.741	3.519	1.472	1.380	0.561	–	0.377	66.805
CI_am_	14.183	13.123	0.895	0.165	0.411	0.517	0.298	–	0.610	69.798
CI_saHCl_	15.058	5.688	2.190	1.724	1.092	1.207	0.433	0.230	0.347	72.031
CI_amHCl_	14.720	10.703	1.162	0.110	0.244	0.817	0.406	0.107	0.494	71.237
CT_a_	15.451	5.510	1.871	3.809	1.279	1.226	0.467	–	0.627	69.760
CT_sa_	13.976	5.088	2.262	3.830	1.186	1.260	0.244	–	0.605	71.549
CT_amsa_	17.354	10.667	2.299	0.569	0.721	0.780	0.521	–	0.496	66.593
CT_am_	8.602	10.015	2.060	0.125	0.037	0.082	0.071	–	0.456	78.552
CT_amsb_	20.780	8.424	2.187	5.755	1.700	2.394	0.505	–	0.522	57.733
CT_amHCl_	17.477	11.084	2.266	–	0.564	0.766	0.094	0.189	0.529	67.031
CT_amsbHCl_	14.909	13.180	0.744	0.170	0.279	0.473	0.066	0.178	0.590	69.410

**Table 2:** Correlation between the normal caustic and summative modules, and the free acidity.

Sample	Normal Caustic Module Si/Al	Summative Module Si(Ti/Fe^III^+Cl)/ Al(Ca/Mg+Na/K)	pH	[H^+^] (×10^–5^ mol/L)
CI_a_	3.892	1.855	5.0	1.000
CI_sa_	2.538	1.464	6.0	0.1000
CI_am_	1.081	1.081	7.0	0.0100
CI_saHCl_	2.647	1.757	4.7	1.9953
CI_amHCl_	1.375	1.342	5.8	0.1585
CT_a_	2.804	1.460	6.5	0.3162
CT_sa_	2.747	1.451	6.2	0.0631
CT_amsa_	1.627	1.520	5.2	0.3162
CT_am_	0.859	1.076	7.0	0.0100
CT_amsb_	2.467	1.251	7.1	0.0080
CT_amHCl_	1.577	1.636	4.5	3.1623
CT_amsbHCl_	1.131	1.159	7.5	0.0031
